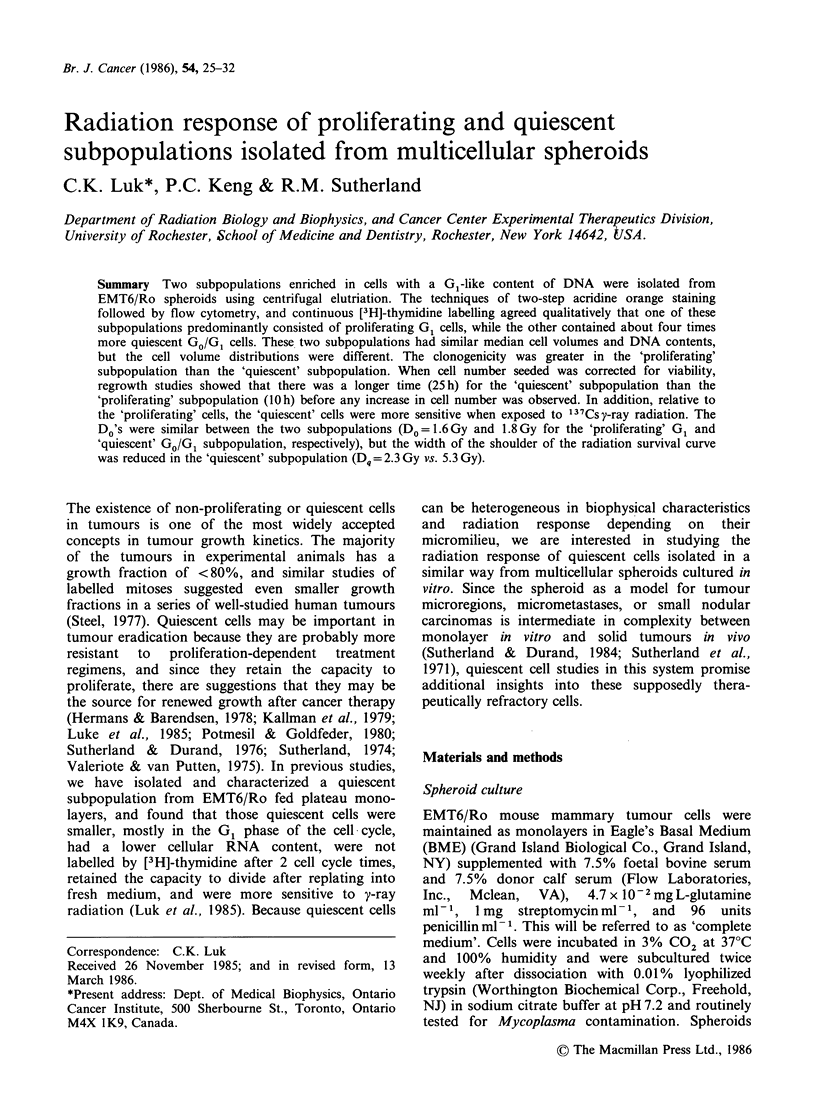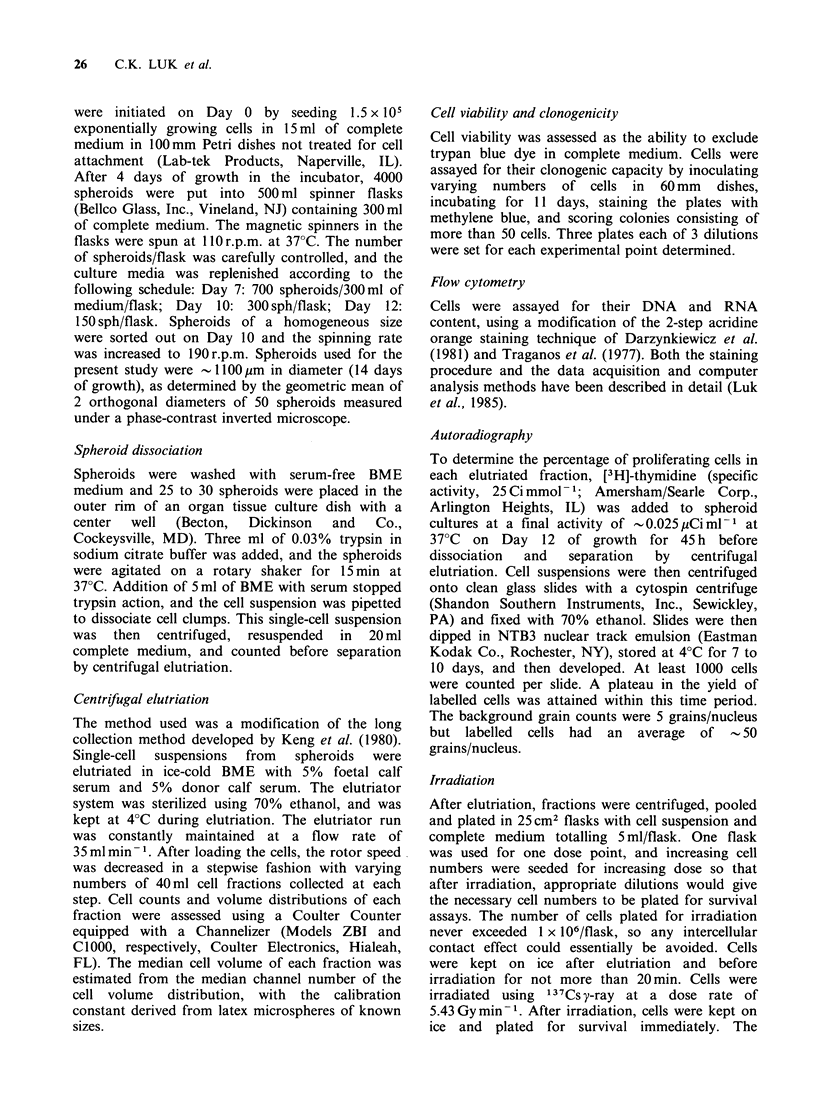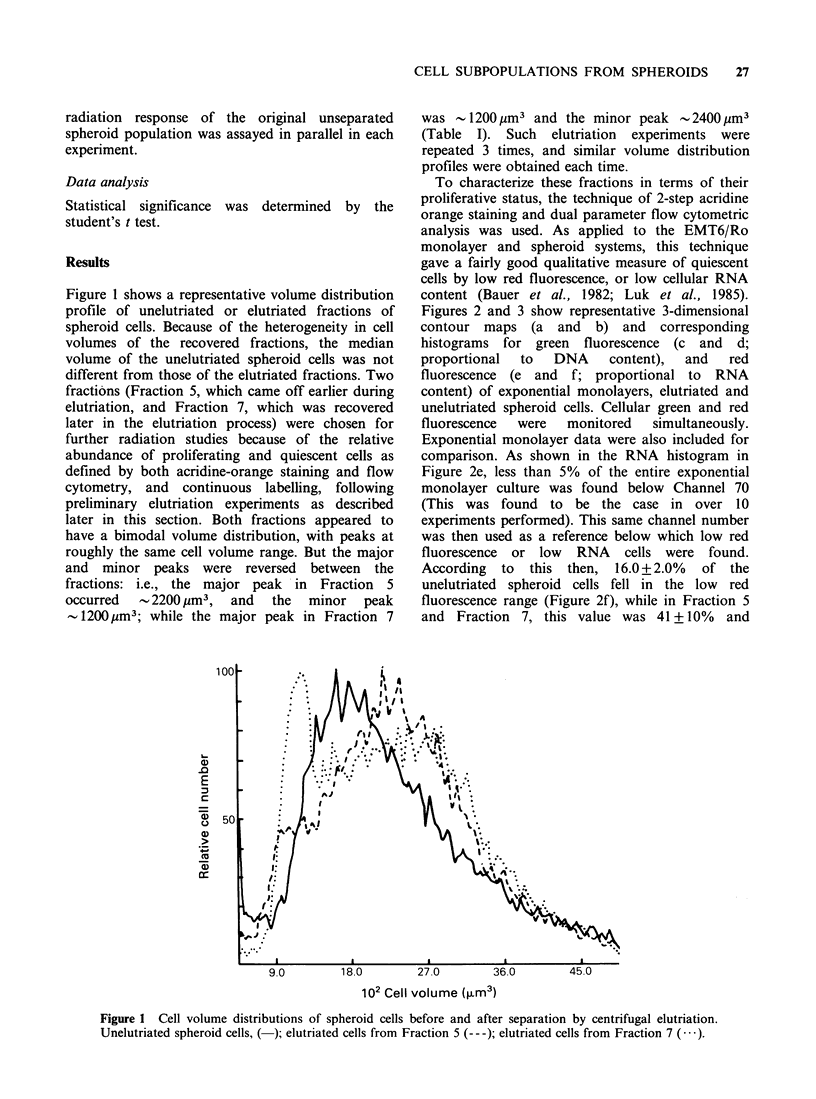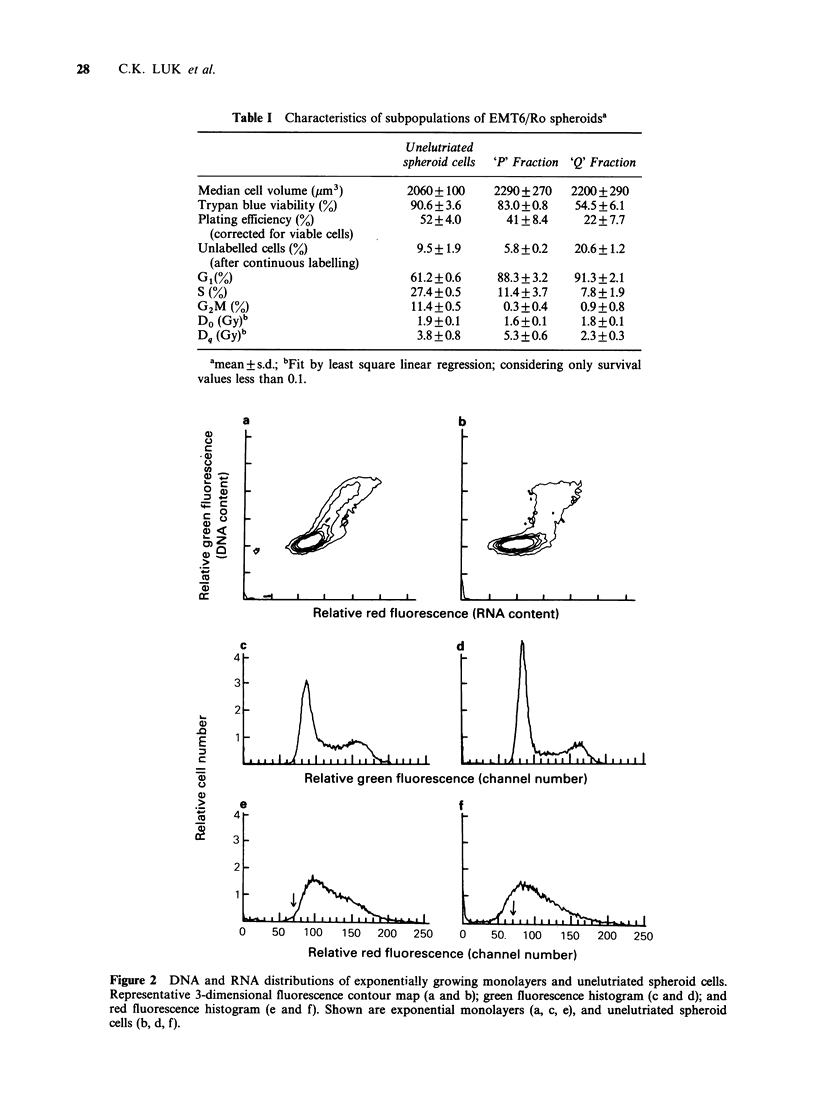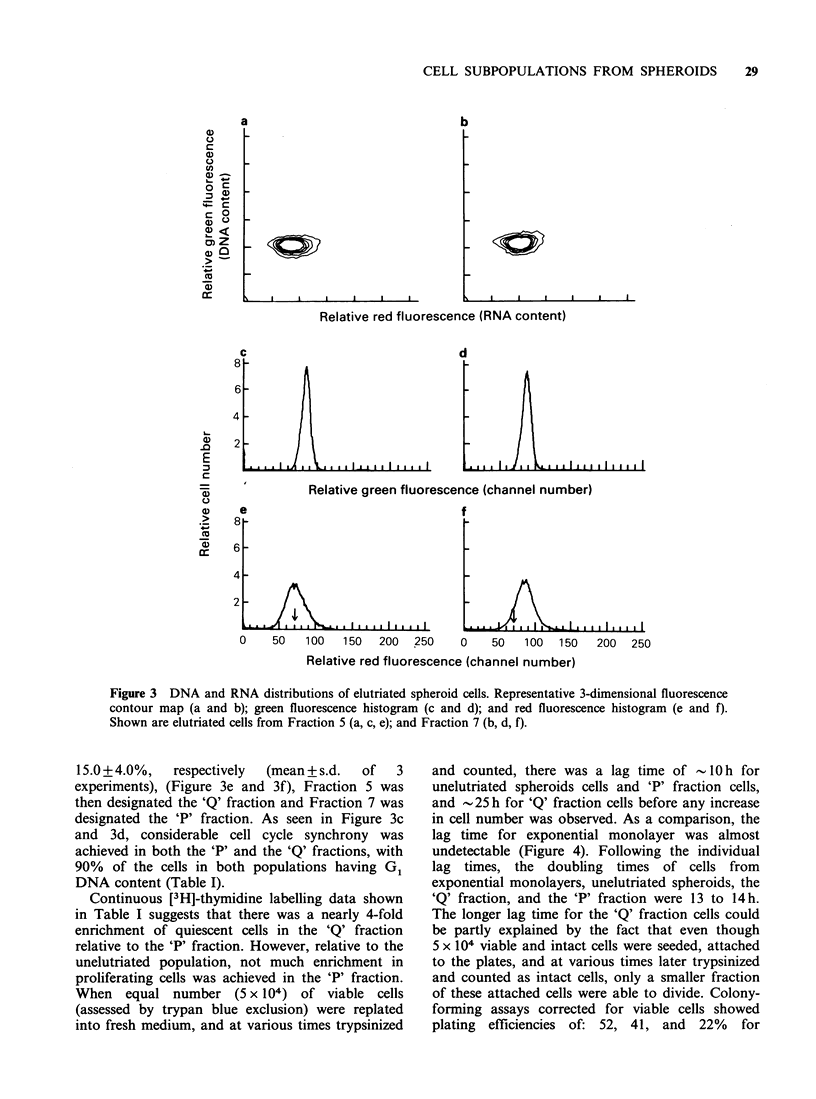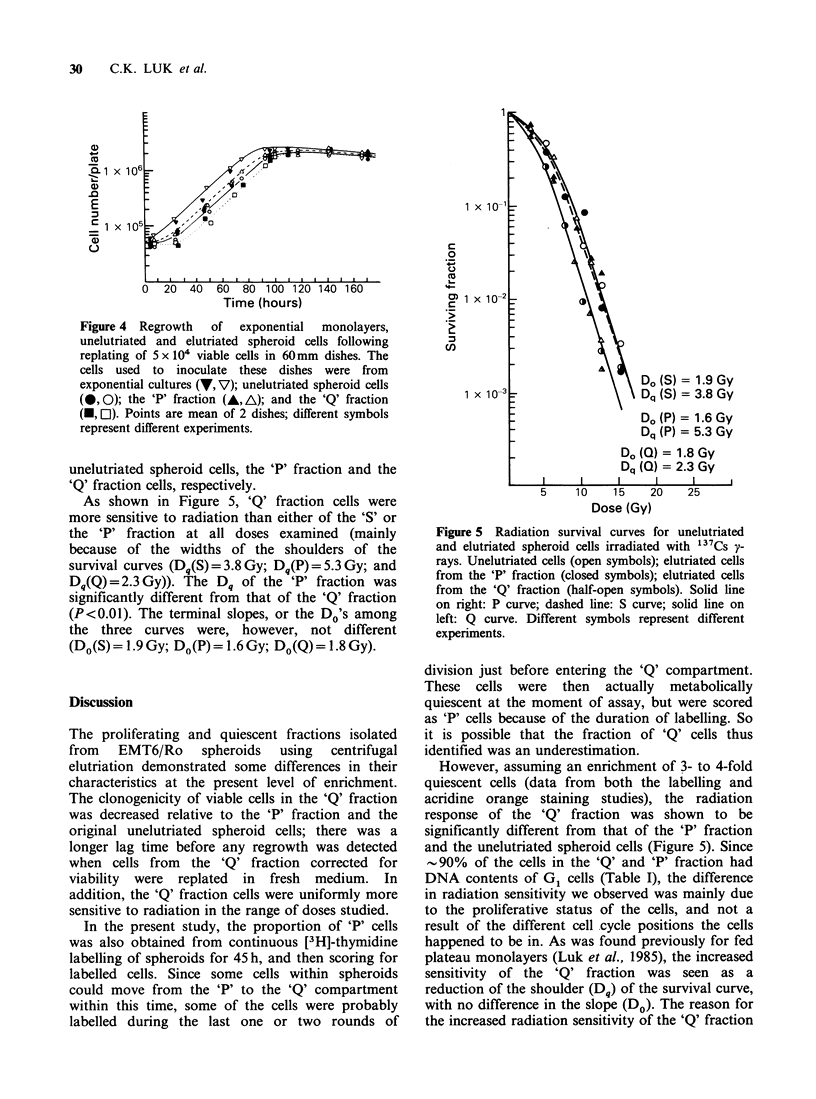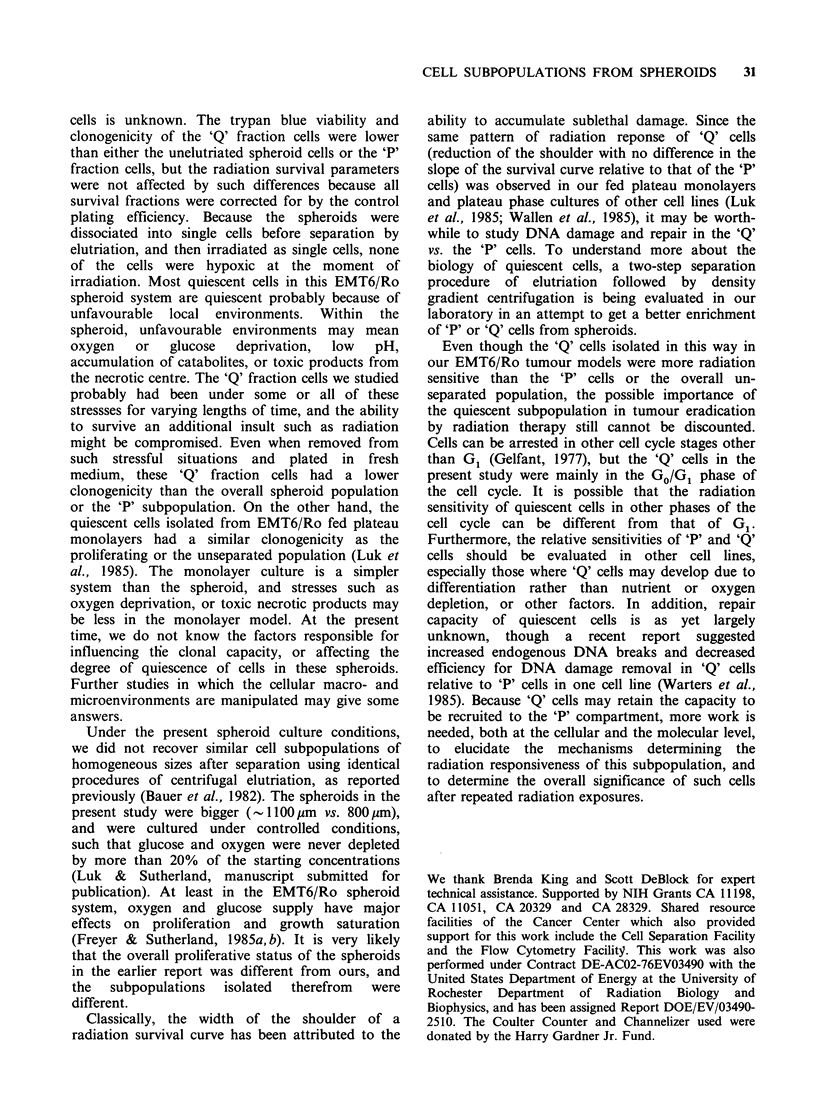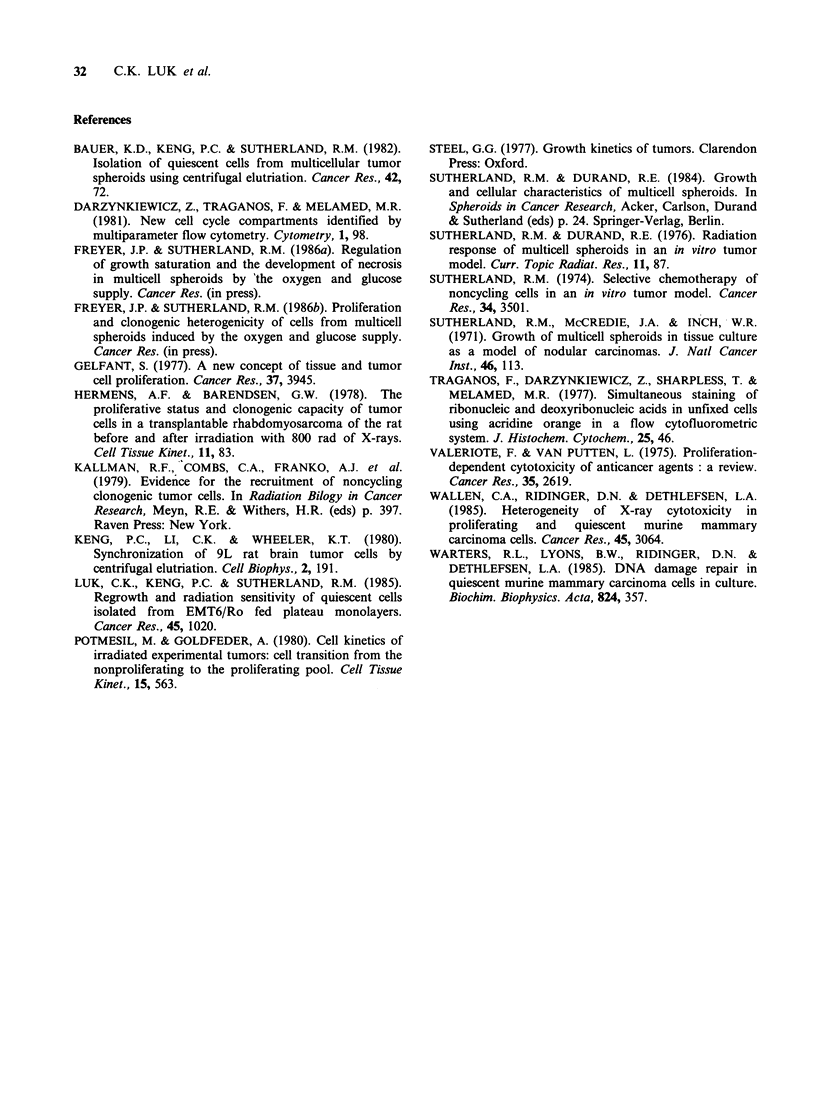# Radiation response of proliferating and quiescent subpopulations isolated from multicellular spheroids.

**DOI:** 10.1038/bjc.1986.148

**Published:** 1986-07

**Authors:** C. K. Luk, P. C. Keng, R. M. Sutherland

## Abstract

Two subpopulations enriched in cells with a G1-like content of DNA were isolated from EMT6/Ro spheroids using centrifugal elutriation. The techniques of two-step acridine orange staining followed by flow cytometry, and continuous [3H]-thymidine labelling agreed qualitatively that one of these subpopulations predominantly consisted of proliferating G1 cells, while the other contained about four times more quiescent G0/G1 cells. These two subpopulations had similar median cell volumes and DNA contents, but the cell volume distributions were different. The clonogenicity was greater in the 'proliferating' subpopulation than the 'quiescent' subpopulation. When cell number seeded was corrected for viability, regrowth studies showed that there was a longer time (25 h) for the 'quiescent' subpopulation than the 'proliferating' subpopulation (10 h) before any increase in cell number was observed. In addition, relative to the 'proliferating' cells, the 'quiescent' cells were more sensitive when exposed to 137Cs gamma-ray radiation. The D0's were similar between the two subpopulations (D0 = 1.6 Gy and 1.8 Gy for the 'proliferating' G1 and 'quiescent' G0/G1 subpopulation, respectively), but the width of the shoulder of the radiation survival curve was reduced in the 'quiescent' subpopulation (Dq = 2.3 Gy vs. 5.3 Gy).


Br. J. Cancer (1986), **54**, 25–32

### Radiation response of proliferating and quiescent subpopulations isolated from multicellular spheroids

C.K. Luk*, P.C. Keng & R.M. Sutherland

*Department of Radiation Biology and Biophysics, and Cancer Center Experimental Therapeutics Division, University of Rochester, School of Medicine and Dentistry, Rochester, New York 14642, USA.*

**Summary** Two subpopulations enriched in cells with a $G_1$-like content of DNA were isolated from EMT6/Ro spheroids using centrifugal elutriation. The techniques of two-step acridine orange staining followed by flow cytometry, and continuous [$^3$H]-thymidine labelling agreed qualitatively that one of these subpopulations predominantly consisted of proliferating $G_1$ cells, while the other contained about four times more quiescent $G_0/G_1$ cells. These two subpopulations had similar median cell volumes and DNA contents, but the cell volume distributions were different. The clonogenicity was greater in the 'proliferating' subpopulation than the 'quiescent' subpopulation. When cell number seeded was corrected for viability, regrowth studies showed that there was a longer time (25 h) for the 'quiescent' subpopulation than the 'proliferating' subpopulation (10 h) before any increase in cell number was observed. In addition, relative to the 'proliferating' cells, the 'quiescent' cells were more sensitive when exposed to $^{137}$Cs $\gamma$-ray radiation. The $D_0$'s were similar between the two subpopulations ($D_0 = 1.6$ Gy and 1.8 Gy for the 'proliferating' $G_1$ and 'quiescent' $G_0/G_1$ subpopulation, respectively), but the width of the shoulder of the radiation survival curve was reduced in the 'quiescent' subpopulation ($D_q = 2.3$ Gy *vs.* 5.3 Gy).

The existence of non-proliferating or quiescent cells in tumours is one of the most widely accepted concepts in tumour growth kinetics. The majority of the tumours in experimental animals has a growth fraction of <80%, and similar studies of labelled mitoses suggested even smaller growth fractions in a series of well-studied human tumours (Steel, 1977). Quiescent cells may be important in tumour eradication because they are probably more resistant to proliferation-dependent treatment regimens, and since they retain the capacity to proliferate, there are suggestions that they may be the source for renewed growth after cancer therapy (Hermans & Barendsen, 1978; Kallman *et al.*, 1979; Luke *et al.*, 1985; Potmesil & Goldfeder, 1980; Sutherland & Durand, 1976; Sutherland, 1974; Valeriote & van Putten, 1975). In previous studies, we have isolated and characterized a quiescent subpopulation from EMT6/Ro fed plateau monolayers, and found that those quiescent cells were smaller, mostly in the $G_1$ phase of the cell cycle, had a lower cellular RNA content, were not labelled by [$^3$H]-thymidine after 2 cell cycle times, retained the capacity to divide after replating into fresh medium, and were more sensitive to $\gamma$-ray radiation (Luk *et al.*, 1985). Because quiescent cells can be heterogeneous in biophysical characteristics and radiation response depending on their micromilieu, we are interested in studying the radiation response of quiescent cells isolated in a similar way from multicellular spheroids cultured *in vitro*. Since the spheroid as a model for tumour microregions, micrometastases, or small nodular carcinomas is intermediate in complexity between monolayer *in vitro* and solid tumours *in vivo* (Sutherland & Durand, 1984; Sutherland *et al.*, 1971), quiescent cell studies in this system promise additional insights into these supposedly therapeutically refractory cells.

Correspondence: C.K. Luk
Received 26 November 1985; and in revised form, 13 March 1986.
*Present address: Dept. of Medical Biophysics, Ontario Cancer Institute, 500 Sherbourne St., Toronto, Ontario M4X 1K9, Canada.

### Materials and methods

### *Spheroid culture*

EMT6/Ro mouse mammary tumour cells were maintained as monolayers in Eagle's Basal Medium (BME) (Grand Island Biological Co., Grand Island, NY) supplemented with 7.5% foetal bovine serum and 7.5% donor calf serum (Flow Laboratories, Inc., Mclean, VA), $4.7 \times 10^{-2}$ mg L-glutamine ml$^{-1}$, 1 mg streptomycin ml$^{-1}$, and 96 units penicillin ml$^{-1}$. This will be referred to as 'complete medium'. Cells were incubated in 3% $CO_2$ at 37°C and 100% humidity and were subcultured twice weekly after dissociation with 0.01% lyophilized trypsin (Worthington Biochemical Corp., Freehold, NJ) in sodium citrate buffer at pH 7.2 and routinely tested for *Mycoplasma* contamination. Spheroids

© The Macmillan Press Ltd., 1986

were initiated on Day 0 by seeding $1.5 \times 10^5$ exponentially growing cells in 15 ml of complete medium in 100 mm Petri dishes not treated for cell attachment (Lab-tek Products, Naperville, IL). After 4 days of growth in the incubator, 4000 spheroids were put into 500 ml spinner flasks (Bellco Glass, Inc., Vineland, NJ) containing 300 ml of complete medium. The magnetic spinners in the flasks were spun at 110 r.p.m. at 37°C. The number of spheroids/flask was carefully controlled, and the culture media was replenished according to the following schedule: Day 7: 700 spheroids/300 ml of medium/flask; Day 10: 300 sph/flask; Day 12: 150 sph/flask. Spheroids of a homogeneous size were sorted out on Day 10 and the spinning rate was increased to 190 r.p.m. Spheroids used for the present study were ~1100 μm in diameter (14 days of growth), as determined by the geometric mean of 2 orthogonal diameters of 50 spheroids measured under a phase-contrast inverted microscope.

*Spheroid dissociation*

Spheroids were washed with serum-free BME medium and 25 to 30 spheroids were placed in the outer rim of an organ tissue culture dish with a center well (Becton, Dickinson and Co., Cockeysville, MD). Three ml of 0.03% trypsin in sodium citrate buffer was added, and the spheroids were agitated on a rotary shaker for 15 min at 37°C. Addition of 5 ml of BME with serum stopped trypsin action, and the cell suspension was pipetted to dissociate cell clumps. This single-cell suspension was then centrifuged, resuspended in 20 ml complete medium, and counted before separation by centrifugal elutriation.

*Centrifugal elutriation*

The method used was a modification of the long collection method developed by Keng *et al.* (1980). Single-cell suspensions from spheroids were elutriated in ice-cold BME with 5% foetal calf serum and 5% donor calf serum. The elutriator system was sterilized using 70% ethanol, and was kept at 4°C during elutriation. The elutriator run was constantly maintained at a flow rate of $35\,ml\,min^{-1}$. After loading the cells, the rotor speed was decreased in a stepwise fashion with varying numbers of 40 ml cell fractions collected at each step. Cell counts and volume distributions of each fraction were assessed using a Coulter Counter equipped with a Channelizer (Models ZBI and C1000, respectively, Coulter Electronics, Hialeah, FL). The median cell volume of each fraction was estimated from the median channel number of the cell volume distribution, with the calibration constant derived from latex microspheres of known sizes.

*Cell viability and clonogenicity*

Cell viability was assessed as the ability to exclude trypan blue dye in complete medium. Cells were assayed for their clonogenic capacity by inoculating varying numbers of cells in 60 mm dishes, incubating for 11 days, staining the plates with methylene blue, and scoring colonies consisting of more than 50 cells. Three plates each of 3 dilutions were set for each experimental point determined.

*Flow cytometry*

Cells were assayed for their DNA and RNA content, using a modification of the 2-step acridine orange staining technique of Darzynkiewicz *et al.* (1981) and Traganos *et al.* (1977). Both the staining procedure and the data acquisition and computer analysis methods have been described in detail (Luk *et al.*, 1985).

*Autoradiography*

To determine the percentage of proliferating cells in each elutriated fraction, [$^3$H]-thymidine (specific activity, $25\,Ci\,mmol^{-1}$; Amersham/Searle Corp., Arlington Heights, IL) was added to spheroid cultures at a final activity of $\sim 0.025\,\mu Ci\,ml^{-1}$ at 37°C on Day 12 of growth for 45 h before dissociation and separation by centrifugal elutriation. Cell suspensions were then centrifuged onto clean glass slides with a cytospin centrifuge (Shandon Southern Instruments, Inc., Sewickley, PA) and fixed with 70% ethanol. Slides were then dipped in NTB3 nuclear track emulsion (Eastman Kodak Co., Rochester, NY), stored at 4°C for 7 to 10 days, and then developed. At least 1000 cells were counted per slide. A plateau in the yield of labelled cells was attained within this time period. The background grain counts were 5 grains/nucleus but labelled cells had an average of ~50 grains/nucleus.

*Irradiation*

After elutriation, fractions were centrifuged, pooled and plated in $25\,cm^2$ flasks with cell suspension and complete medium totalling 5 ml/flask. One flask was used for one dose point, and increasing cell numbers were seeded for increasing dose so that after irradiation, appropriate dilutions would give the necessary cell numbers to be plated for survival assays. The number of cells plated for irradiation never exceeded $1 \times 10^6$/flask, so any intercellular contact effect could essentially be avoided. Cells were kept on ice after elutriation and before irradiation for not more than 20 min. Cells were irradiated using $^{137}Cs\,\gamma$-ray at a dose rate of $5.43\,Gy\,min^{-1}$. After irradiation, cells were kept on ice and plated for survival immediately. The radiation response of the original unseparated spheroid population was assayed in parallel in each experiment.

*Data analysis*

Statistical significance was determined by the student's *t* test.

### Results

Figure 1 shows a representative volume distribution profile of unelutriated or elutriated fractions of spheroid cells. Because of the heterogeneity in cell volumes of the recovered fractions, the median volume of the unelutriated spheroid cells was not different from those of the elutriated fractions. Two fractions (Fraction 5, which came off earlier during elutriation, and Fraction 7, which was recovered later in the elutriation process) were chosen for further radiation studies because of the relative abundance of proliferating and quiescent cells as defined by both acridine-orange staining and flow cytometry, and continuous labelling, following preliminary elutriation experiments as described later in this section. Both fractions appeared to have a bimodal volume distribution, with peaks at roughly the same cell volume range. But the major and minor peaks were reversed between the fractions: i.e., the major peak in Fraction 5 occurred ~2200 $\mu m^3$, and the minor peak ~1200 $\mu m^3$; while the major peak in Fraction 7 was ~1200 $\mu m^3$ and the minor peak ~2400 $\mu m^3$ (Table I). Such elutriation experiments were repeated 3 times, and similar volume distribution profiles were obtained each time.

To characterize these fractions in terms of their proliferative status, the technique of 2-step acridine orange staining and dual parameter flow cytometric analysis was used. As applied to the EMT6/Ro monolayer and spheroid systems, this technique gave a fairly good qualitative measure of quiescent cells by low red fluorescence, or low cellular RNA content (Bauer *et al.,* 1982; Luk *et al.,* 1985). Figures 2 and 3 show representative 3-dimensional contour maps (a and b) and corresponding histograms for green fluorescence (c and d; proportional to DNA content), and red fluorescence (e and f; proportional to RNA content) of exponential monolayers, elutriated and unelutriated spheroid cells. Cellular green and red fluorescence were monitored simultaneously. Exponential monolayer data were also included for comparison. As shown in the RNA histogram in Figure 2e, less than 5% of the entire exponential monolayer culture was found below Channel 70 (This was found to be the case in over 10 experiments performed). This same channel number was then used as a reference below which low red fluorescence or low RNA cells were found. According to this then, 16.0±2.0% of the unelutriated spheroid cells fell in the low red fluorescence range (Figure 2f), while in Fraction 5 and Fraction 7, this value was 41±10% and

**Figure 1** Cell volume distributions of spheroid cells before and after separation by centrifugal elutriation. Unelutriated spheroid cells, (—); elutriated cells from Fraction 5 (---); elutriated cells from Fraction 7 (···).

**Table I** Characteristics of subpopulations of EMT6/Ro spheroids[a]

| | *Unelutriated spheroid cells* | *'P' Fraction* | *'Q' Fraction* |
|---|---|---|---|
| Median cell volume ($\mu m^3$) | 2060±100 | 2290±270 | 2200±290 |
| Trypan blue viability (%) | 90.6±3.6 | 83.0±0.8 | 54.5±6.1 |
| Plating efficiency (%) (corrected for viable cells) | 52±4.0 | 41±8.4 | 22±7.7 |
| Unlabelled cells (%) (after continuous labelling) | 9.5±1.9 | 5.8±0.2 | 20.6±1.2 |
| $G_1$(%) | 61.2±0.6 | 88.3±3.2 | 91.3±2.1 |
| S (%) | 27.4±0.5 | 11.4±3.7 | 7.8±1.9 |
| $G_2M$ (%) | 11.4±0.5 | 0.3±0.4 | 0.9±0.8 |
| $D_0$ (Gy)[b] | 1.9±0.1 | 1.6±0.1 | 1.8±0.1 |
| $D_q$ (Gy)[b] | 3.8±0.8 | 5.3±0.6 | 2.3±0.3 |

[a]mean±s.d.; [b]Fit by least square linear regression; considering only survival values less than 0.1.

**Figure 2** DNA and RNA distributions of exponentially growing monolayers and unelutriated spheroid cells. Representative 3-dimensional fluorescence contour map (a and b); green fluorescence histogram (c and d); and red fluorescence histogram (e and f). Shown are exponential monolayers (a, c, e), and unelutriated spheroid cells (b, d, f).

**Figure 3** DNA and RNA distributions of elutriated spheroid cells. Representative 3-dimensional fluorescence contour map (a and b); green fluorescence histogram (c and d); and red fluorescence histogram (e and f). Shown are elutriated cells from Fraction 5 (a, c, e); and Fraction 7 (b, d, f).

15.0±4.0%, respectively (mean±s.d. of 3 experiments), (Figure 3e and 3f), Fraction 5 was then designated the 'Q' fraction and Fraction 7 was designated the 'P' fraction. As seen in Figure 3c and 3d, considerable cell cycle synchrony was achieved in both the 'P' and the 'Q' fractions, with 90% of the cells in both populations having $G_1$ DNA content (Table I).

Continuous [$^3$H]-thymidine labelling data shown in Table I suggests that there was a nearly 4-fold enrichment of quiescent cells in the 'Q' fraction relative to the 'P' fraction. However, relative to the unelutriated population, not much enrichment in proliferating cells was achieved in the 'P' fraction. When equal number ($5\times10^4$) of viable cells (assessed by trypan blue exclusion) were replated into fresh medium, and at various times trypsinized and counted, there was a lag time of ~10 h for unelutriated spheroids cells and 'P' fraction cells, and ~25 h for 'Q' fraction cells before any increase in cell number was observed. As a comparison, the lag time for exponential monolayer was almost undetectable (Figure 4). Following the individual lag times, the doubling times of cells from exponential monolayers, unelutriated spheroids, the 'Q' fraction, and the 'P' fraction were 13 to 14 h. The longer lag time for the 'Q' fraction cells could be partly explained by the fact that even though $5\times10^4$ viable and intact cells were seeded, attached to the plates, and at various times later trypsinized and counted as intact cells, only a smaller fraction of these attached cells were able to divide. Colony-forming assays corrected for viable cells showed plating efficiencies of: 52, 41, and 22% for

**Figure 4** Regrowth of exponential monolayers, unelutriated and elutriated spheroid cells following replating of $5 \times 10^4$ viable cells in 60mm dishes. The cells used to inoculate these dishes were from exponential cultures (▼, ▽); unelutriated spheroid cells (●, ○); the 'P' fraction (▲, △); and the 'Q' fraction (■, □). Points are mean of 2 dishes; different symbols represent different experiments.

**Figure 5** Radiation survival curves for unelutriated and elutriated spheroid cells irradiated with $^{137}Cs$ $\gamma$-rays. Unelutriated cells (open symbols); elutriated cells from the 'P' fraction (closed symbols); elutriated cells from the 'Q' fraction (half-open symbols). Solid line on right: P curve; dashed line: S curve; solid line on left: Q curve. Different symbols represent different experiments.

unelutriated spheroid cells, the 'P' fraction and the 'Q' fraction cells, respectively.

As shown in Figure 5, 'Q' fraction cells were more sensitive to radiation than either of the 'S' or the 'P' fraction at all doses examined (mainly because of the widths of the shoulders of the survival curves ($D_q$(S) = 3.8 Gy; $D_q$(P) = 5.3 Gy; and $D_q$(Q) = 2.3 Gy)). The $D_q$ of the 'P' fraction was significantly different from that of the 'Q' fraction ($P < 0.01$). The terminal slopes, or the $D_0$'s among the three curves were, however, not different ($D_0$(S) = 1.9 Gy; $D_0$(P) = 1.6 Gy; $D_0$(Q) = 1.8 Gy).

### Discussion

The proliferating and quiescent fractions isolated from EMT6/Ro spheroids using centrifugal elutriation demonstrated some differences in their characteristics at the present level of enrichment. The clonogenicity of viable cells in the 'Q' fraction was decreased relative to the 'P' fraction and the original unelutriated spheroid cells; there was a longer lag time before any regrowth was detected when cells from the 'Q' fraction corrected for viability were replated in fresh medium. In addition, the 'Q' fraction cells were uniformly more sensitive to radiation in the range of doses studied.

In the present study, the proportion of 'P' cells was also obtained from continuous [$^3$H]-thymidine labelling of spheroids for 45 h, and then scoring for labelled cells. Since some cells within spheroids could move from the 'P' to the 'Q' compartment within this time, some of the cells were probably labelled during the last one or two rounds of division just before entering the 'Q' compartment. These cells were then actually metabolically quiescent at the moment of assay, but were scored as 'P' cells because of the duration of labelling. So it is possible that the fraction of 'Q' cells thus identified was an underestimation.

However, assuming an enrichment of 3- to 4-fold quiescent cells (data from both the labelling and acridine orange staining studies), the radiation response of the 'Q' fraction was shown to be significantly different from that of the 'P' fraction and the unelutriated spheroid cells (Figure 5). Since ~90% of the cells in the 'Q' and 'P' fraction had DNA contents of $G_1$ cells (Table I), the difference in radiation sensitivity we observed was mainly due to the proliferative status of the cells, and not a result of the different cell cycle positions the cells happened to be in. As was found previously for fed plateau monolayers (Luk *et al.*, 1985), the increased sensitivity of the 'Q' fraction was seen as a reduction of the shoulder ($D_q$) of the survival curve, with no difference in the slope ($D_0$). The reason for the increased radiation sensitivity of the 'Q' fraction cells is unknown. The trypan blue viability and clonogenicity of the 'Q' fraction cells were lower than either the unelutriated spheroid cells or the 'P' fraction cells, but the radiation survival parameters were not affected by such differences because all survival fractions were corrected for by the control plating efficiency. Because the spheroids were dissociated into single cells before separation by elutriation, and then irradiated as single cells, none of the cells were hypoxic at the moment of irradiation. Most quiescent cells in this EMT6/Ro spheroid system are quiescent probably because of unfavourable local environments. Within the spheroid, unfavourable environments may mean oxygen or glucose deprivation, low pH, accumulation of catabolites, or toxic products from the necrotic centre. The 'Q' fraction cells we studied probably had been under some or all of these stressses for varying lengths of time, and the ability to survive an additional insult such as radiation might be compromised. Even when removed from such stressful situations and plated in fresh medium, these 'Q' fraction cells had a lower clonogenicity than the overall spheroid population or the 'P' subpopulation. On the other hand, the quiescent cells isolated from EMT6/Ro fed plateau monolayers had a similar clonogenicity as the proliferating or the unseparated population (Luk *et al.*, 1985). The monolayer culture is a simpler system than the spheroid, and stresses such as oxygen deprivation, or toxic necrotic products may be less in the monolayer model. At the present time, we do not know the factors responsible for influencing the clonal capacity, or affecting the degree of quiescence of cells in these spheroids. Further studies in which the cellular macro- and microenvironments are manipulated may give some answers.

Under the present spheroid culture conditions, we did not recover similar cell subpopulations of homogeneous sizes after separation using identical procedures of centrifugal elutriation, as reported previously (Bauer *et al.*, 1982). The spheroids in the present study were bigger ($\sim 1100\,\mu$m *vs.* $800\,\mu$m), and were cultured under controlled conditions, such that glucose and oxygen were never depleted by more than 20% of the starting concentrations (Luk & Sutherland, manuscript submitted for publication). At least in the EMT6/Ro spheroid system, oxygen and glucose supply have major effects on proliferation and growth saturation (Freyer & Sutherland, 1985*a,b*). It is very likely that the overall proliferative status of the spheroids in the earlier report was different from ours, and the subpopulations isolated therefrom were different.

Classically, the width of the shoulder of a radiation survival curve has been attributed to the ability to accumulate sublethal damage. Since the same pattern of radiation reponse of 'Q' cells (reduction of the shoulder with no difference in the slope of the survival curve relative to that of the 'P' cells) was observed in our fed plateau monolayers and plateau phase cultures of other cell lines (Luk *et al.*, 1985; Wallen *et al.*, 1985), it may be worthwhile to study DNA damage and repair in the 'Q' *vs.* the 'P' cells. To understand more about the biology of quiescent cells, a two-step separation procedure of elutriation followed by density gradient centrifugation is being evaluated in our laboratory in an attempt to get a better enrichment of 'P' or 'Q' cells from spheroids.

Even though the 'Q' cells isolated in this way in our EMT6/Ro tumour models were more radiation sensitive than the 'P' cells or the overall unseparated population, the possible importance of the quiescent subpopulation in tumour eradication by radiation therapy still cannot be discounted. Cells can be arrested in other cell cycle stages other than $G_1$ (Gelfant, 1977), but the 'Q' cells in the present study were mainly in the $G_0/G_1$ phase of the cell cycle. It is possible that the radiation sensitivity of quiescent cells in other phases of the cell cycle can be different from that of $G_1$. Furthermore, the relative sensitivities of 'P' and 'Q' cells should be evaluated in other cell lines, especially those where 'Q' cells may develop due to differentiation rather than nutrient or oxygen depletion, or other factors. In addition, repair capacity of quiescent cells is as yet largely unknown, though a recent report suggested increased endogenous DNA breaks and decreased efficiency for DNA damage removal in 'Q' cells relative to 'P' cells in one cell line (Warters *et al.*, 1985). Because 'Q' cells may retain the capacity to be recruited to the 'P' compartment, more work is needed, both at the cellular and the molecular level, to elucidate the mechanisms determining the radiation responsiveness of this subpopulation, and to determine the overall significance of such cells after repeated radiation exposures.

We thank Brenda King and Scott DeBlock for expert technical assistance. Supported by NIH Grants CA 11198, CA 11051, CA 20329 and CA 28329. Shared resource facilities of the Cancer Center which also provided support for this work include the Cell Separation Facility and the Flow Cytometry Facility. This work was also performed under Contract DE-AC02-76EV03490 with the United States Department of Energy at the University of Rochester Department of Radiation Biology and Biophysics, and has been assigned Report DOE/EV/03490-2510. The Coulter Counter and Channelizer used were donated by the Harry Gardner Jr. Fund.

### References

BAUER, K.D., KENG, P.C. & SUTHERLAND, R.M. (1982). Isolation of quiescent cells from multicellular tumor spheroids using centrifugal elutriation. *Cancer Res.*, **42**, 72.

DARZYNKIEWICZ, Z., TRAGANOS, F. & MELAMED, M.R. (1981). New cell cycle compartments identified by multiparameter flow cytometry. *Cytometry*, **1**, 98.

FREYER, J.P. & SUTHERLAND, R.M. (1986*a*). Regulation of growth saturation and the development of necrosis in multicell spheroids by the oxygen and glucose supply. *Cancer Res.* (in press).

FREYER, J.P. & SUTHERLAND, R.M. (1986*b*). Proliferation and clonogenic heterogenicity of cells from multicell spheroids induced by the oxygen and glucose supply. *Cancer Res.* (in press).

GELFANT, S. (1977). A new concept of tissue and tumor cell proliferation. *Cancer Res.*, **37**, 3945.

HERMENS, A.F. & BARENDSEN, G.W. (1978). The proliferative status and clonogenic capacity of tumor cells in a transplantable rhabdomyosarcoma of the rat before and after irradiation with 800 rad of X-rays. *Cell Tissue Kinet.*, **11**, 83.

KALLMAN, R.F., COMBS, C.A., FRANKO, A.J. *et al.* (1979). Evidence for the recruitment of noncycling clonogenic tumor cells. In *Radiation Bilogy in Cancer Research*, Meyn, R.E. & Withers, H.R. (eds) p. 397. Raven Press: New York.

KENG, P.C., LI, C.K. & WHEELER, K.T. (1980). Synchronization of 9L rat brain tumor cells by centrifugal elutriation. *Cell Biophys.*, **2**, 191.

LUK, C.K., KENG, P.C. & SUTHERLAND, R.M. (1985). Regrowth and radiation sensitivity of quiescent cells isolated from EMT6/Ro fed plateau monolayers. *Cancer Res.*, **45**, 1020.

POTMESIL, M. & GOLDFEDER, A. (1980). Cell kinetics of irradiated experimental tumors: cell transition from the nonproliferating to the proliferating pool. *Cell Tissue Kinet.*, **15**, 563.

STEEL, G.G. (1977). Growth kinetics of tumors. Clarendon Press: Oxford.

SUTHERLAND, R.M. & DURAND, R.E. (1984). Growth and cellular characteristics of multicell spheroids. In *Spheroids in Cancer Research*, Acker, Carlson, Durand & Sutherland (eds) p. 24. Springer-Verlag, Berlin.

SUTHERLAND, R.M. & DURAND, R.E. (1976). Radiation response of multicell spheroids in an *in vitro* tumor model. *Curr. Topic Radiat. Res.*, **11**, 87.

SUTHERLAND, R.M. (1974). Selective chemotherapy of noncycling cells in an *in vitro* tumor model. *Cancer Res.*, **34**, 3501.

SUTHERLAND, R.M., McCREDIE, J.A. & INCH, W.R. (1971). Growth of multicell spheroids in tissue culture as a model of nodular carcinomas. *J. Natl Cancer Inst.*, **46**, 113.

TRAGANOS, F., DARZYNKIEWICZ, Z., SHARPLESS, T. & MELAMED, M.R. (1977). Simultaneous staining of ribonucleic and deoxyribonucleic acids in unfixed cells using acridine orange in a flow cytofluorometric system. *J. Histochem. Cytochem.*, **25**, 46.

VALERIOTE, F. & VAN PUTTEN, L. (1975). Proliferation-dependent cytotoxicity of anticancer agents : a review. *Cancer Res.*, **35**, 2619.

WALLEN, C.A., RIDINGER, D.N. & DETHLEFSEN, L.A. (1985). Heterogeneity of X-ray cytotoxicity in proliferating and quiescent murine mammary carcinoma cells. *Cancer Res.*, **45**, 3064.

WARTERS, R.L., LYONS, B.W., RIDINGER, D.N. & DETHLEFSEN, L.A. (1985). DNA damage repair in quiescent murine mammary carcinoma cells in culture. *Biochim. Biophysics. Acta*, **824**, 357.